# Strontium Retention of Calcium Zirconium Aluminate Cement Paste Studied by NMR, XRD and SEM-EDS

**DOI:** 10.3390/ma13102366

**Published:** 2020-05-21

**Authors:** Dominika Madej

**Affiliations:** Faculty of Materials Science and Ceramics, AGH University of Science and Technology, al. A. Mickiewicza 30, 30-059 Krakow, Poland; dmadej@agh.edu.pl

**Keywords:** Ca_7_ZrAl_6_O_18_, ^27^Al MAS NMR, Sr-rich (Sr,C)_3_AH_6_, cement hydration, refractories, immobilization of radioactive Sr

## Abstract

This work concerns the hydration mechanism of calcium zirconium aluminate as a ternary compound appearing in the CaO-Al_2_O_3_-ZrO_2_ diagram besides the calcium aluminates commonly used as the main constitutes of calcium aluminate cements (CACs). Moreover, a state-of-the-art approach towards significant changes in hydraulic properties was implemented for the first time in this work, where the effect of structural modification on the hydration behavior of calcium zirconium aluminate was proved by XRD, ^27^Al MAS NMR and SEM-EDS. The substitution of Sr^2+^ for Ca^2+^ in the Ca_7_ZrAl_6_O_18_ lattice decreases the reactivity of Sr-substituted Ca_7_ZrAl_6_O_18_ in the presence of water. Since the original cement grains remain unhydrated up to 3 h (Ca_7_ZrAl_6_O_18_) or 72 h (Sr_1.25_Ca_5.75_ZrAl_6_O_18_) of curing period in the hardened cement paste structures, strontium can be considered as an inhibition agent for cement hydration. The complete conversion from anhydrous ^27^Al^IV^ to hydrated ^27^Al^VI^ species was achieved during the first 24 h (Ca_7_ZrAl_6_O_18_) or 7 d(Sr_1.25_Ca_5.75_ZrAl_6_O_18_) of hydration. Simultaneously, the chemical shift in the range of octahedral aluminum from ca. 4 ppm to ca. 6 ppm was attributed to the transformation of the hexagonal calcium aluminate hydrates and Sr-rich (Sr,C)_3_AH_6_ hydrate into the cubic phase Ca-rich (Sr,C)_3_AH_6_ or pure C_3_AH_6_ in the hardened Sr-doped cement paste at the age of 7 d. The same ^27^Al NMR chemical shift was detected at the age of 24 h for the reference hardened undoped Ca_7_ZrAl_6_O_18_ cement paste.

## 1. Introduction

Calcium aluminate cements (CACs) [1,2,3] and other related cementitious systems [4,5,6,7] are believed to play an important role in a wide range of specialist are as from some construction areas and civil engineering, to refractory materials industry, due to their ability to gain strength rapidly in the initial days after casting and to withstand aggressive environments and high temperatures. The temperature–time weight ratio of water-to-cement (the w/c ratio) dependencies of the cement hydration processes have been widely investigated so far and are presented elsewhere in detail [8,9,10]. It must be made clear at this stage that both temperature and a weight ratio of water-to-cement (the w/c ratio) affect the performances of calcium aluminate cements, especially at an early age. The phase composition, microstructure and other properties of the cement pastes can easily be designed by choosing appropriate curing parameters. Nevertheless, upon curing of CAC-based paste at not adequately controlled, ambient conditions, these materials are very sensitive to humidity, CO_2_, temperature and time. Due to the fact of not being able to predict their long-term behavior makes them practically not suitable for constructions but suitable for building chemistry. Nevertheless, the high-early-heat and high-early-strength gain makes CACs attractive, especially in the winter months and/or when rapid repairs are needed. The hydration product formation in CACs containing mainly monocalcium aluminate (CaAl_2_O_4_; CA) and monocalcium di aluminate (CaAl_4_O_7_; CA_2_) is characterized by the dependence of C−A−H* (C = CaO, A = Al_2_O_3_, H = H_2_O) phases on temperature. At a temperature below ca. 15 °C, calcium aluminates are hydrated to form metastable CAH_10_. At room temperature, the hydration process proceeds through the formation of other metastable phases C_2_AH_8_ and/or C_4_AH_13–19_ (hexagonal hydrates). At higher temperatures (above 28 °C), the metastable hydrates will spontaneously convert to the cubic hydrogarnet C_3_AH_6_. Furthermore, according to insights into hydration of CACs, the hydration products during long hydration shall be presented as follows: C_3_AH_6_ + AH_3_. The 10-year results, which have been added to existing results on the hydration of calcium aluminate compounds, are of interest as showing the CaO-Al_2_O_3_-ZrO_2_ [4,6,11] as an interesting alternative to CACs-based materials. This can be attributed to the fact, that this category of special cement which contains both hydraulic phases (CA, CA_2_, C_12_A_7_ and C_7_A_3_Z*), and Zr-bearing compounds (CZ and Z) [4] is synthesized by one environmentally friendly and effective technological process. This fact makes the attention-getter of a C-A-Z cements’ users more important than those of other CACs users and worth considering from the point of view of economics. The principal interest here is concerned with the C_7_A_3_Z area including crystal structure, hydration behavior of C_7_A_3_Z and its solid solutions with SrO and BaO, hydration products and carbonation processes in cement paste structures [12,13]. Special interest was given to strontium. Because strontium is chemically closely related to calcium, it is easily introduced as a natural substitute for calcium in aluminate phases. Strontium has been shown to affect the hydration behavior of calcium zirconium aluminate cement [5,13]. Furthermore, strontium-containing cements attract the attention of materials scientists due to their higher refractory properties, increased resistance to thermal shock, increased resistance to chemical aggressive solutions and possible applications as special binders for shielding constructions in nuclear power plants [14]. Although this approach is unique, there are other approaches to affecting the hydration behavior of aluminates-bearing CACs and related materials. Results from other studies indicate that the metal chloride and nitrate salts are known in CAC science as agents affecting the CAC hydration behavior [15,16]. The exact nature of this behavior is different for alkali metal salts (NaCl, KCl, RbCl, CsCl, LiCl), alkaline earth metal salts (MgCl_2_∙6H_2_O, CaCl_2_, SrCl_2_∙6H_2_O and BaCl_2_∙2H_2_O) and transition metal salts (MnCl_2_∙H_2_O, CoCl_2_∙6H_2_O, CuCl_2_∙2H_2_O and ZnCl_2_). Generally, the addition of alkali metal salts accelerates the setting time of CAC, whereas the transition metal salts can be widely used as retarders. The effect of alkaline earth metal salts on setting the behavior of CAC is determined by the amount of addition [15]. Another approach in the acceleration effect is the addition of chlorides and nitrates of Li(I), Cr(III), Zn(II) and Cr(VI) (chromate), whereas Pb(II) and Cu(II) retarded the hydration [16].

Many various techniques including XRD, FT-IR, Raman spectroscopy, DSC-TG-EGA(MS), SEM-EDS, isothermal calorimetry and EIS were being accepted in this research. It is also well known that solid-state nuclear magnetic resonance (ssNMR) spectroscopy is an effective tool for the characterization of cement paste at the atomic scale in different stages of its aging. The application of NMR to monitor the progress of CACs hydration has a long history and can be found elsewhere [17,18,19,20]. In cement chemistry, four-fold coordinated (tetrahedral)^IV^Al sites and six-fold coordinated (octahedral)^VI^Al sites typically resonate within the regions 50–80 ppm and 0–20 ppm, respectively. The resonances assigned to five-coordinated ^V^Al and highly distorted tetrahedral Al environments have been observed in the region 20–50 ppm [18,21,22]. For example, the ^27^Al MAS NMR spectrum of CA shows a peak maximum at ca. 80 ppm and a shoulder at 76 ppm due to six crystal ographically different aluminum tetrahedral sites [19,23,24,25]. For another example, the ^27^Al MAS NMR spectrum of CA_2_ exhibits the peaks between 50 and 75 ppm due to the two distinct ^27^Al environments [18,25]. On the other hand, calcium aluminate hydrates (CAH_10_, C_2_AH_8_, C_4_AH_13-19_, C_3_AH_6_), originating from the hydration process of CAC clinker phases, are octahedrally coordinated Al(OH)_6_ and cause a ^27^Al MAS NMR signal at about 0 ppm. Hence, ^27^Al MAS NMR provides the relative changes of tetrahedral and octahedral Al sites of the hydrating cement paste at different stages of the curing. The resonance position from an octahedral ^27^Al NMR signal varies slightly depending on the types of calcium aluminate hydrates present [19]. The resonance at ca. 12.36 ppm is due to tricalcium aluminate hexahydrate C_3_AH_6_ [18,20], at ca. 10.2 ppm due to CAH_10_ and C_4_AH_13_ [18,19,20] and at ca. 10.3 ppm due to C_2_AH_8_ [17].

The aim of this work is to implement the NMR technique to monitor the progress of hydration of both undoped- and strontium-doped calcium zirconium aluminate cement. Hence, the influence of structural modification with SrO on the hydraulic activity of Ca_7_ZrAl_6_O_18_ phase has been demonstrated. This work fills in a significant gap in the literature on the exploiting ssNMR spectroscopy to probe the early stages of hydration of new types of cements belonging to the CaO-Al_2_O_3_-ZrO_2_ and CaO-SrO-Al_2_O_3_-ZrO_2_ systems.

## 2. Experimental Procedure

### 2.1. Synthesis and Phase Identification

Low-cost synthesis of Ca_7_ZrAl_6_O_18_, Sr_1.25_Ca_5.75_Al_6_O_18_ and SrAl_2_O_4_ cements via the solid-state reactive sintering technique was employed using the stoichiometric amount of the cationic ratios of Ca:Zr:Al = 7:1:6, Sr:Ca:Zr:Al = 1.25:5.75:1:6 and Sr:Al = 1:2, respectively. Strontium was incorporated into Ca_7_ZrAl_6_O_18_ by replacing 1.25 atoms of calcium, since the maximum level of doping was experimentally established. Both Ca_7_ZrAl_6_O_18_ and SrAl_2_O_4_ cements were synthesized as reference materials to obtain pure C-A-H and Sr-A-H phases. Starting raw materials were reagent-grade CaCO_3_ (99.9%, POCH), SrCO_3_ (98.0%, Merck), Al_2_O_3_ (99.0%, Acros Organics) and ZrO_2_ (98.5%, Acros Organics). The 100 g mixtures of substrates were homogenized for 2 h, pressed into cylindrical pellets at pressures of 50 MPa and calcined at 1000 °C for 10 h (Sr-free sample) or at 1300 °C for 10 h (Sr-containing samples) in air. Phase pure cement clinker minerals were made from mixtures of prereacted powders via grinding, pressing and sintering at 1420 °C (Ca_7_ZrAl_6_O_18_ and Sr_1.25_Ca_5.75_Al_6_O_18_) and 1550 °C (SrAl_2_O_4_) for 20 h in air.

Phase identification for the sintered samples was carried out using X-ray diffraction (XRD, PANalytical, Malvern PANalytical, Malvern, UK) on a ProPANalytical X’Pert X-ray diffractometer, with Cu Kα radiation (λ = 0.15418 nm), with 0.02° per step and 3s time per step (2theta range from 5° to 45°).

The NMR spectra were recordedat room temperature on Bruker Avance III 400WB (9.4T) spectrometer, (Bruker BioSpin, Rheinstetten, Germany) using 4 mm MAS (Magic Angle Spinning), dual-channel (1H/BB) probe-head, operating at a resonance frequency of 104.26 MHz for ^27^Al. The sample was spun at a MAS frequency of 8 kHz in the rotors made of zirconium dioxide (4 mm). 32 K data points and 1024 scans FIDs were accumulated with a Single Pulse Excitation (SPE) pulse sequence using the observed 90° pulse (^27^Al) set at 6.0 us with a relaxation delay of 200 ms. Note no proton decoupling was applied during the experiment. Prior to Fourier transformation, the data were zero-filled twice and 80 Hz apodization filter was applied. The ^27^Al chemical shifts were referenced using a sample of AlCl_3_·6H_2_O in 1M solution as an external reference (0 ppm).

The microstructures of fracture surfaces of hydrated cement pastes were investigated using a scanning electron microscope (SEM, FEI Nova Nano SEM 200, Kyoto, Japan.). The chemical compositions of the samples were determined with electron-probe microanalysis using an energy-dispersive X-ray spectrometer (EDAX, Sapphire Si(Li) EDS detector, Mahwah, NJ, USA).

### 2.2. Preparation and Treatment of Cement Paste

Comparison study of hydration characteristics between Ca_7_ZrAl_6_O_18,_ Sr_1.25_Ca_5.75_Al_6_O_18_ and SrAl_2_O_4_ cements were determined for cement pastes prepared with water-to-cement (w/c) ratios of 1.0 or 0.5. The water-to-cement ratio of 0.5 was applied to achieve plastic properties without any undesirable sedimentation of neat SrAl_2_O_4_ paste. Whereas, both Ca_7_ZrAl_6_O_18_ and Sr_1.25_Ca_5.75_Al_6_O_18_ cements require w/c = 1.0 to obtain the well-homogenized cement pastes without any undesirable phenomena of sedimentation. The cement powders which were obtained by grinding the sintered pellets and necessary mass of water were mixed together in a glass beaker to obtain three homogeneous neat cement pastes. Each neat cement paste was then placed in a polyethylene bag and sealed until 14 d at 50 °C. According to Litwinek and Madej [5], the optimal synthesis temperature for C_3_AH_6_ from different precursors through hydration is suggested to be 50 °C. Moreover, this period for curing cement pastes was accepted to attain the maximum degree of hydration, as concluded from the previous studies [13]. Moreover, as it was previously mentioned by Garcés et al. [26] and Zhang et al. [27], at temperatures as high as 60 °C, only the cubic phase and the gibbsite appear in the calcium aluminates-based cement pastes. At 24 h and 7 d, the microstructure of cement pastes was investigated by SEM. Acetone quenching was used to stop hydration at 15 min, 0.5 h, 1 h, 2 h, 3 h, 24 h, 48 h, 72 h, 7 d and 14 d (Table 1). The use of cold acetone, aiming to withdraw free water and inhibit further reactions within cement paste is known from the Ref. [28]. Cold acetone is related to acetone stored under laboratory conditions. Quenched pastes were characterized by XRD and ^27^Al MAS NMR (Bruker BioSpin, Rheinstetten, Germany), according to the procedures presented in Section 2.1.

## 3. Results and Discussion

### 3.1. X-ray Diffraction Analysis of Special Cements Hydration

According to X-ray diffraction analysis, the cement clinkers synthesized by the solid-state reactive sintering technique were all crystalline, single-phase aluminate phases Ca_7_ZrAl_6_O_18_, Sr-doped Ca_7_ZrAl_6_O_18_ and SrAl_2_O_4_. The positions of the characteristic diffraction peaks in the XRD patterns of the Ca_7_ZrAl_6_O_18_ (Sample C) and SrAl_2_O_4_ (Sample B) cement clinkers have a strong agreement with the standards JCPDS No. 98-018-2622 and JCPDS No. 98-016-0296, respectively. This confirms that the powders are mainly composed of Ca_7_ZrAl_6_O_18_ and SrAl_2_O_4_, respectively. As expected, the slight shift in diffraction peaks towards lower 2θ value in the XRD pattern of Sr_1.25_Ca_5.75_ZrAl_6_O_18_ cement clinker confirms an increase in lattice parameter of Sr-doped Ca_7_ZrAl_6_O_18_ solid solution. Since no secondary phases containing Sr were detected, Sr was recognized as fully incorporated into the Ca_7_ZrAl_6_O_18_ structure.

**Figure 1:**
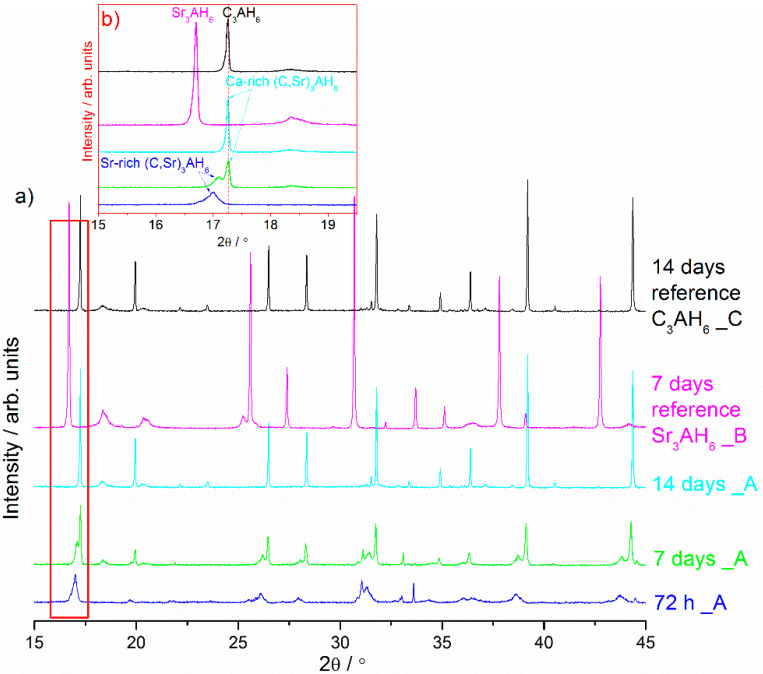

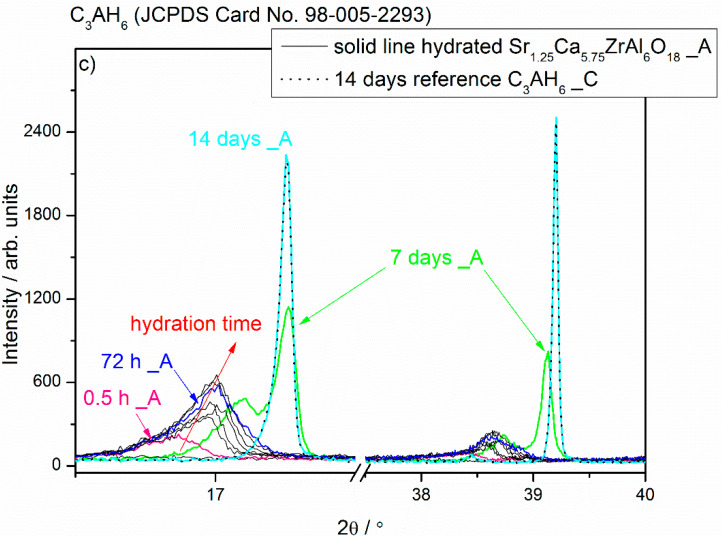
X-ray diffraction patterns of the Sr_1.25_Ca_5.75_ZrAl_6_O_18_ cement paste (Sample A) at different curing periods. (**a**,**b**) contains lines of two reference materials, i.e., Sr_3_AH_6_ synthesized through hydration from SrAl_2_O_4_ cement (Sample B) and C_3_AH_6_ synthesized through hydration from Ca_7_ZrAl_6_O_18_ cement (Sample C). (**c**) contains a line (dot line) of the reference C_3_AH_6_ formed at 14 d (Sample C).

**Figure 2:**
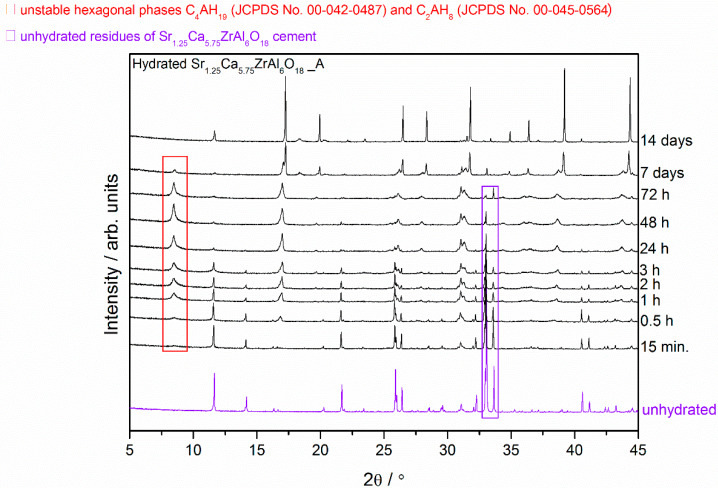
X-ray diffraction patterns of the Sr_1.25_Ca_5.75_ZrAl_6_O_18_ cement paste (Sample A) at different curing periods from 15 min to 14 d compared with the XRD pattern of the unhydrated compound.

**Figure 3:**
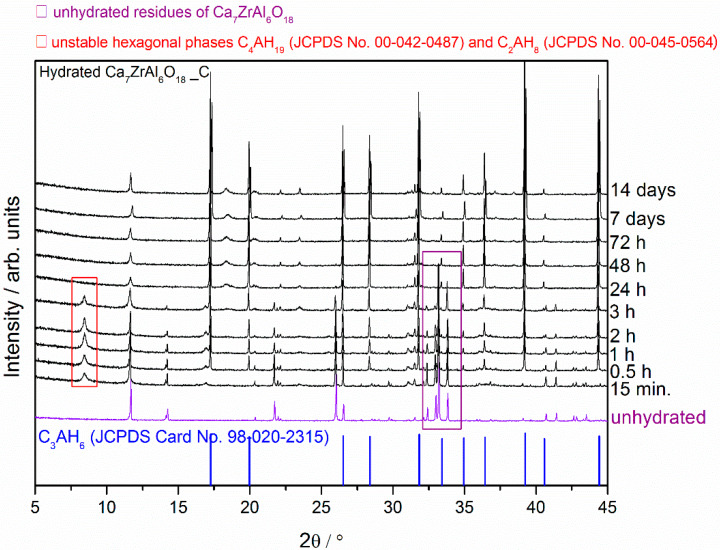
X-ray diffraction patterns of the Ca_7_ZrAl_6_O_18_ cement paste (Sample C) at different curing periods from 15 min to 14 d compared with the XRD pattern of the unhydrated compound.

Compared with the XRD patterns of unhydrated phases, the decreasing intensity of peaks corresponding to Sr_1.25_Ca_5.75_ZrAl_6_O_18_ and Ca_7_ZrAl_6_O_18_, and new peaks of low intensity corresponding to hydration products formation were recorded using XRD (Figure 1, Figure 2 and Figure 3). A low degree of hydrates crystallinity was indicated by a poor XRD pattern, especially in the early stage of hydration. Moreover, the hydration processes result in a corresponding change in the XRD patterns of the initial cement clinker phases and formation of amorphous material, besides the crystalline hydrates, as it can be concluded from the severe intensity reduction and peaks broadening in the XRD pattern.

The results of powder X-ray diffraction patterns evaluation show the progress of Sr_1.25_Ca_5.75_ZrAl_6_O_18_ hydration in cement paste between 15 min and 14 d of curing which are more or less similar at an early stage of hydration (up to 72 h) Figure 1a–c. The XRD profiles of the cement pastes hydrated between 0.5 and 72 h demonstrate diffraction peak at ca. 17.00° 2θ which can now be interpreted as belonging to Sr-rich (Sr,C)_3_AH_6_ hydrate (Figure 1c). Hence, this XRD peak located at the position of 2θ = 17.00 which belongs to Sr-rich (Sr,C)_3_AH_6_ needs to be considered between the reference C_3_AH_6_ synthesized through Ca_7_ZrAl_6_O_18_ hydration and other reference sample Sr_3_AH_6_ synthesized from SrAl_2_O_4_ precursor through hydration. As is evident from this figure, the formation of the intermediate Sr-rich (Sr,C)_3_AH_6_ hydrate precedes the formation of the stable Ca-rich (Sr,C)_3_AH_6_ hydrate at 7 d of curing. In this sample, two isostructural compounds with a hydrogarnet type crystal lattice were present. The position of the lower-intensity XRD line at ca. 17.00° 2θ is situated between lines belonging to pure phases Sr_3_AH_6_ (Sample B) and C_3_AH_6_ (Sample C). The second position of the higher intensity XRD line at ca. 17.26° 2θ is similar to that found for reference C_3_AH_6_ (Figure 1a–c). In addition, it is worth noting that the Sr-rich (Sr,C)_3_AH_6_ exists in the hardened cement paste between 0.5 h and 7 d of curing. This phase disappeared after longer curing times and became replaced by Ca-rich (Sr,C)_3_AH_6_ or C_3_AH_6_. This work has successfully shown the existence of the solid solution of strontium in the tricalcium hydrate C_3_AH_6_ lattice by direct verification using XRD. By reason of structural modification of C_3_AH_6_ through ionic substitution, the lattice parameter of the cubic phase was increased and the slight shift in XRD peaks belonging to (Sr,C)_3_AH_6_ solid solution towards lower 2θ value was observed (Figure 1b). This increase in the lattice parameter was due to the size of the ionic radius of Sr^2+^ (132 pm) which is bigger than the ionic radius of Ca^2+^ (114 pm).

A brief summary of XRD results is given as Figure 2 and Figure 3. This overview XRD spectra recorded from the Sr_1.25_Ca_5.75_ZrAl_6_O_18_ cement paste (Sample A) at different curing periods from 15 min to 14 d showed a progressive reduction in the peaks associated with Sr_1.25_Ca_5.75_ZrAl_6_O_18_ due to its hydration process, which led to the formation of hydration products (Figure 2). The cement paste at the age of 15 min is a mixture of the unhydrated phase and amorphous or poorly crystalline hexagonal hydrates, whereas the cement paste at the age between 0.5 h and 72 h contained a mixture of the Sr-rich (Sr,C)_3_AH_6_ cubic phase, hexagonal hydrates and the still unhydrated residues of the Sr_1.25_Ca_5.75_ZrAl_6_O_18_ cement grains. It should be noted that the positions of the XRD peaks of C_4_AH_19_ (JCPDS No. 00-042-0487; h k l = 0 0 6, d = 10.64350 Å, 2θ = 8.301°, I = 100%) are coincident well with those belonging to C_2_AH_8_ (JCPDS No. 00-045-0564, h k l = 0 0 6, d = 10.81270 Å, 2θ = 8.170°, I = 100%). Therefore, it is often difficult to clearly differentiate between C_4_AH_19_ and C_2_AH_8_ in the XRD patterns, as is clearly demonstrated with a red rectangle (□) in Figure 2. However, at 7d second adjacent cubic phase, Ca-rich (Sr,C)_3_AH_6_ or pure C_3_AH_6_, exists together with the initially formed cubic phase Sr-rich (Sr,C)_3_AH_6_ and some residues of the hexagonal hydrates. As a general trend at the age of 14 d, XRD pattern of cement paste achieved profile similar to that of pure C_3_AH_6_ (Figure 1c and Figure 2) without any metastable hydrates and unhydrated cement residues, i.e., unhydrated cement clinker mineral Sr_1.25_Ca_5.75_ZrAl_6_O_18_.

The overview XRD spectra recorded from the reference Ca_7_ZrAl_6_O_18_ cement paste (Sample C) at different curing periods from 15 min to 14 d is shown in Figure 3. The hexagonal hydrates exist with the still unhydrated residues of the Ca_7_ZrAl_6_O_18_ in cement paste between 15 min and 3 h of curing period, whereas C_3_AH_6_ hydrated phase is formed at the curing age of 0.5 h. At the age of 24 h, the XRD pattern of the cement pastes exhibits profile similar to that of pure C_3_AH_6_ without any traces of unhydrated cement particles Ca_7_ZrAl_6_O_18_ and metastable hydrates.

From the X-ray diffraction results, it seems obvious that strontium doping affects the hydration behavior of the cement clinker mineral phase Ca_7_ZrAl_6_O_18_, and leads to changes in the hydration products properties. There is a relationship between the proportion of residual unhydrated cement particles and the properties of the particular cement clinker mineral phases involved. After 24 h of curing at 50 °C, where the hardened cement paste (Sample C) consists primarily of C_3_AH_6_, the original Ca_7_ZrAl_6_O_18_ cement particles are no longer evident. In the Sr-doping of Ca_7_ZrAl_6_O_18_ case, there is inhibition of hydration, and the Sr_1.25_Ca_5.75_ZrAl_6_O_18_ cementitious particles exist in the hardened cement paste up to 72 h (Sample A). This material would need to cure over 72 h to reach complete hydration. Hence, XRD data for 0.5 h–7 d materials containing strontium indicates the appearance of additional peaks adjacent to each of the reference C_3_AH_6_ lines caused by the presence of an additional Sr-rich cubic phase. These results confirmed the strontium retention by calcium zirconium aluminate cement paste through the chemical bonding to C-A-H in the hydrated phase. The presence of strontium in the C-A-H matrix is also known to delay the transformation of hexagonal hydrates into the cubic phases.

### 3.2. Ex-Situ ^27^Al NMR Study of the Hydration Reaction at 50 °C

**Figure 4:**
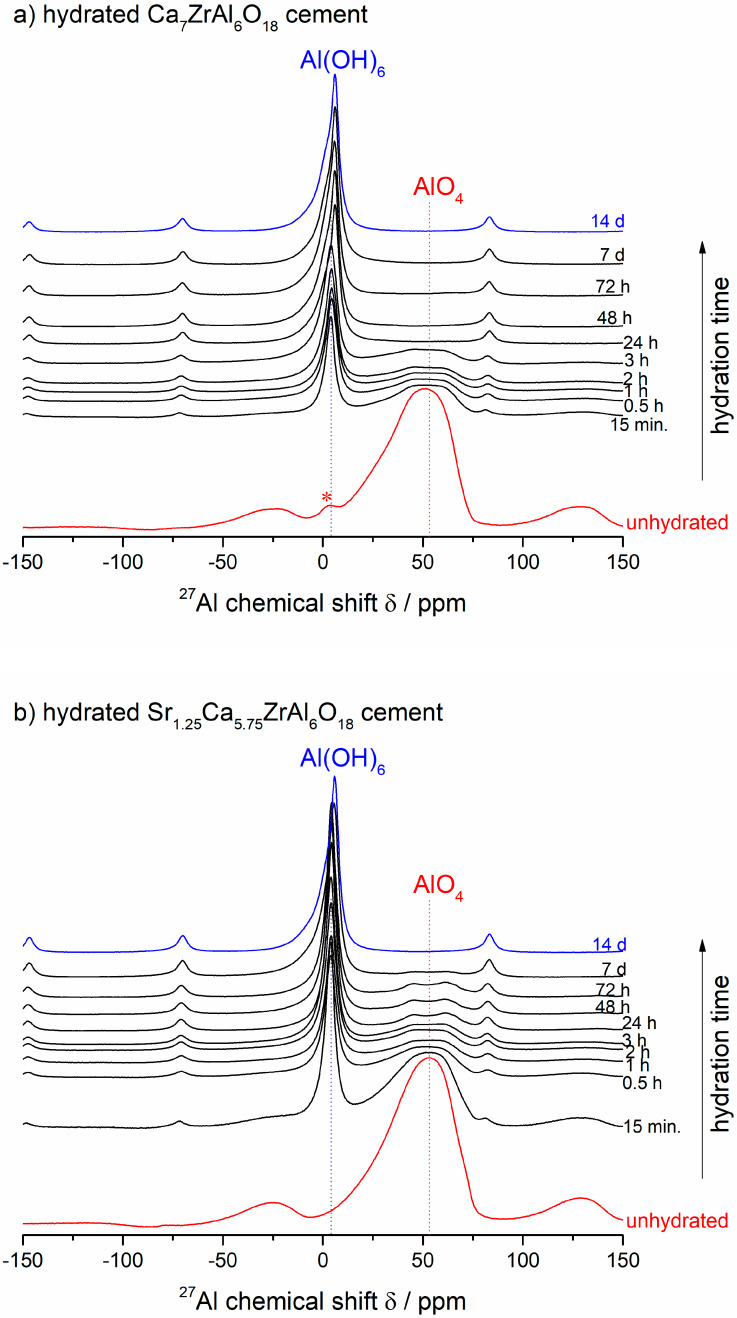

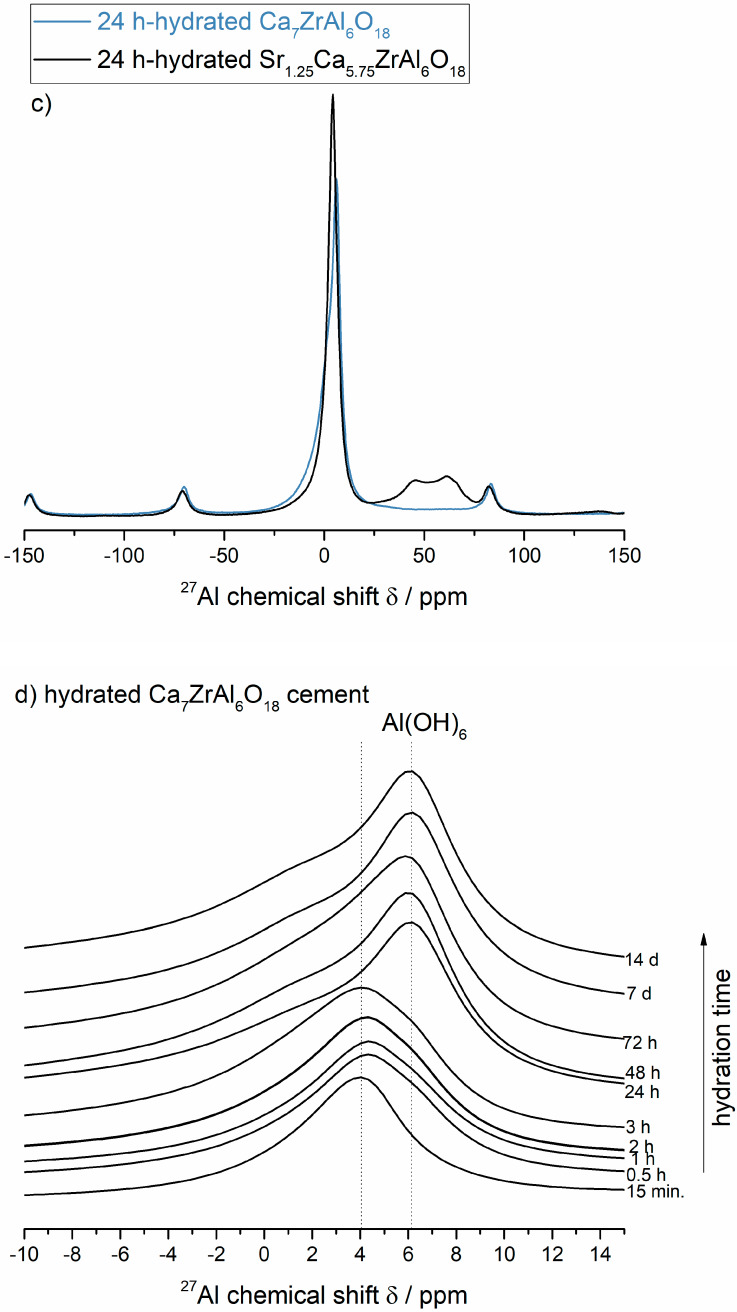

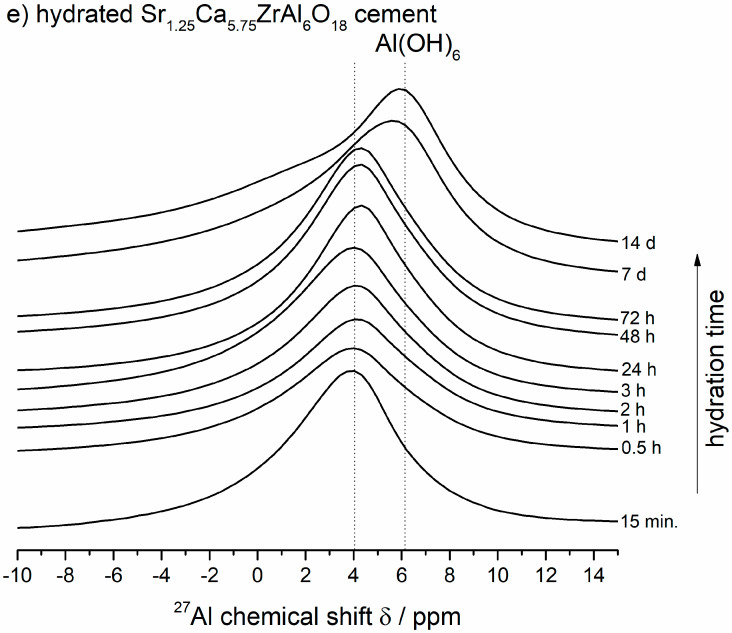
(**a**,**b**) The room-temperature ^27^Al MASNMR spectra of unhydrated Ca_7_ZrAl_6_O_18_ (Sample C) and Sr_1.25_Ca_5.75_ZrAl_6_O_18_ (Sample A) cements, and their products of hydration formed between 15 min and 14 d at 50 °C. The arrow means the changes according to the direction of increasing hydration time. (**c**) A comparison of NMR spectra of both cements at 24 h of hydration. (**d**,**e**) The lines at ca. 4–6 ppm for octahedrally coordinated lattice Al^3+^ in calcium aluminate hydrates.

Figure 4a,b presents the ^27^Al NMR spectra of synthesized Ca_7_ZrAl_6_O_18_ and Sr-doped Ca_7_ZrAl_6_O_18_ cements together with their products of hydration. The ^27^Al MASNMR spectra of the starting unhydrated samples are shown by the red lines. The intense and broad peak at ca. 50 ppm is due to Ca_7_ZrAl_6_O_18_, which consists of orientationally disordered six AlO_4_ tetrahedra linked together by sharing corners, to form [Al_6_O_18_] rings [29]. The ^27^Al MAS NMR spectra of the hydrated samples during the first 15 min all contain peaks near 4 ppm due to ^VI^Al in the cement hydration reaction products (amorphous or poorly crystalline hexagonal hydrates C_4_AH_19_ or C_2_AH_8_) (Figure 4a,b).

For the partially reacted samples, the signals are in the ca. 4 ppm and ca. 46–61 ppm ranges due to both hydrates and unhydrated reactants, respectively (Figure 4c). For the totally hydrated (and converted) samples, all of the signalsarein the ca. 6 ppm range, consistent with total conversion of Al from tetrahedral coordination in the unhydrated Ca_7_ZrAl_6_O_18_ and Sr_1.25_Ca_5.75_ZrAl_6_O_18_ cements to octahedral coordination [19] in the final hydrates formed at 24 h (undoped cement) or 7 d (Sr-doped cement), as expected from previous works on the calcium aluminate cement hydration processes investigated by solid-state^27^Al MAS NMR studies [19,25]. The maximum for the ^VI^Al peak alters slightly depending on the calcium aluminate hydrates present [18,19]. For the undoped Ca_7_ZrAl_6_O_18_ cement hydrated between 15 min and 3 h, in which the detected crystalline hydrates are mainly hexagonal phases, the peak maximum is at ca. 4 ppm and shifts to 6 ppm for this cement hydrated between 24 h and 14 d (Figure 4d), which by XRD contain cubic C_3_AH_6_ as the predominant phase. This type of shift is delayed up to 7 d in the hydrated Sr-doped cement (Figure 4e), where Ca-rich (Sr,C)_3_AH_6_ or pure C_3_AH_6_ begin to form. Hence, it can be summarized that the chemical shift occurring at ca. 4 ppm was due to octahedrally coordinated framework aluminum atoms in Sr-rich (Sr,C)_3_AH_6_ (Sr_1.25_Ca_5.75_ZrAl_6_O_18_ cement paste), poorly crystalline C_3_AH_6_ (Ca_7_ZrAl_6_O_18_ cement paste) and hexagonal hydrates (both cement pastes) formed at an early stage of hydration. The chemical shift occurring at ca. 6 ppm was due to octahedrally coordinated framework aluminum atoms in Ca-rich (Sr,C)_3_AH_6_ or pure C_3_AH_6_ formed in the totally reacted Sr-doped sample, as it can be referenced in for pure C_3_AH_6_ formed in the reference fully hydrated and converted undoped cement paste. It is worth discussing that the maximum for the Al(6) peak belonging to C-A-H phases varies slightly from data presented in Ref. [18,19]. In those works, and many others, the peak maximum belonging to C_3_AH_6_ was located at about 12 ppm, whereas the peak maxima belonging to hexagonal hydrates were located at ca. 10–11 ppm.

### 3.3. Microstructural Studies on the Hydrated Sr_1.25_Ca_5.75_ZrAl_6_O_18_Cement Paste

**Figure 5:**
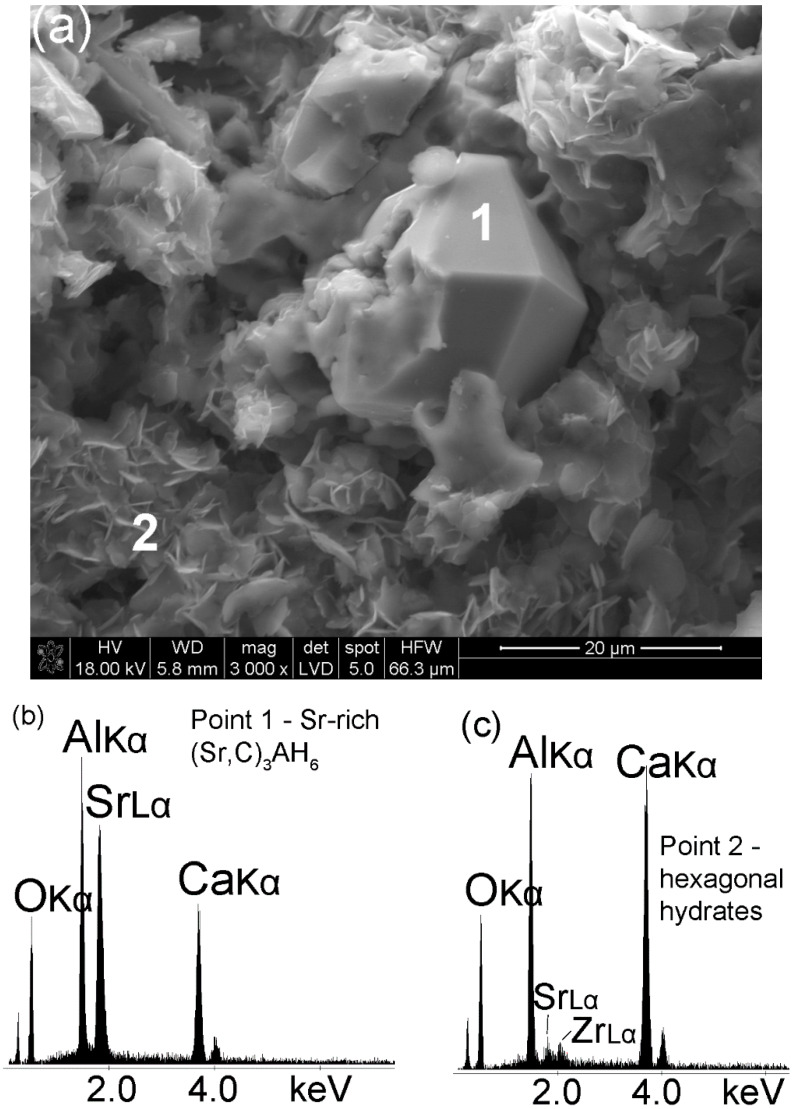
SEM image of the hydrated Sr_1.25_Ca_5.75_ZrAl_6_O_18_ cement (Sample A) at the age of 24 h (**a**). Spot 1–2 EDS analysis; (**b**,**c**) EDS spectra of the sample in the microarea 1 (Sr-rich (Sr,C)_3_AH_6_) and 2 (hexagonal calcium aluminate hydrates), respectively.

The development of Sr_1.25_Ca_5.75_ZrAl_6_O_18_ cement paste microstructure in time can directly be linked to the evolution of phase composition presented in Section 3.1 and Section 3.2. Figure 5 shows the typical microstructure of this cement paste fragment after 24 h hydration at 50 °C. The presence of Sr in Sr_1.25_Ca_5.75_ZrAl_6_O_18_ clinker affects formation of Sr-rich (Sr,C)_3_AH_6_ (Figure 5a—point 1) with a cubic/isometric crystal form [30,31]. The EDS spectrum presenting intensity vs. energy of the detected X-ray clearly identifies the peaks of Sr, Ca, Al and O (Figure 5b). The EDS spectrum from the hexagonal irregular flakes is shown in Figure 5c. The Ca, Al and O peaks are mainly due to C-A-H phases. EDS intensity ratio of calcium and aluminum peaks indicates the presence of C_4_AH_19_ hydrate rather than C_2_AH_8_ hydrate.

**Figure 6:**
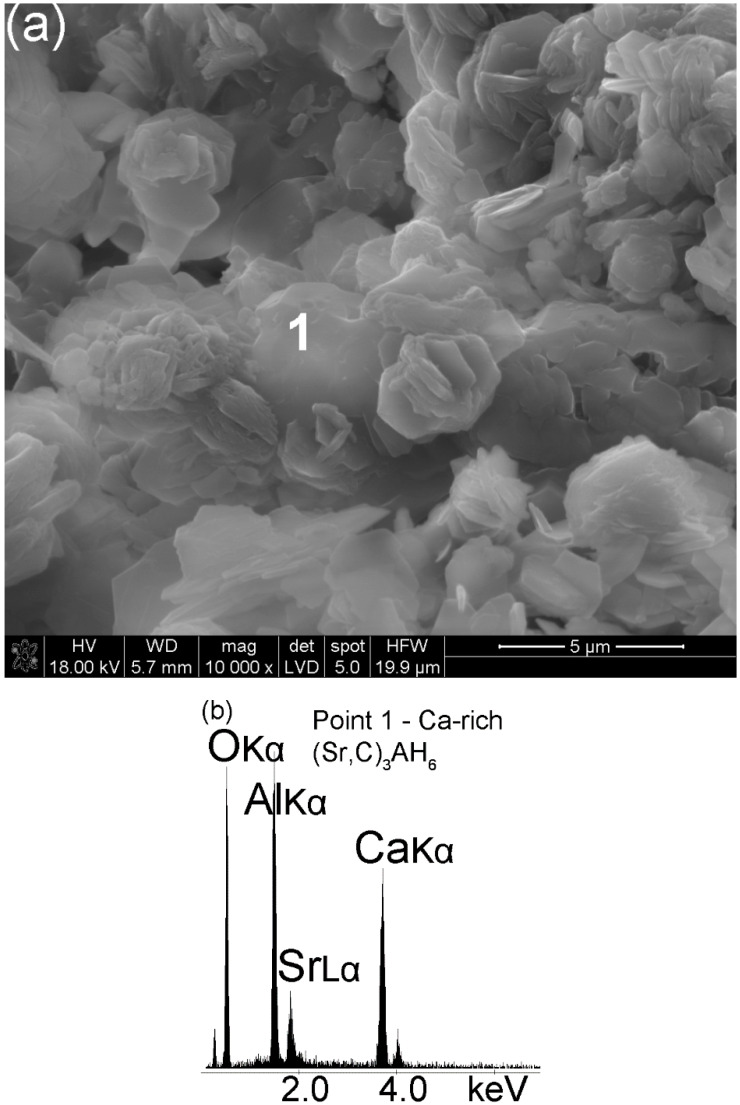

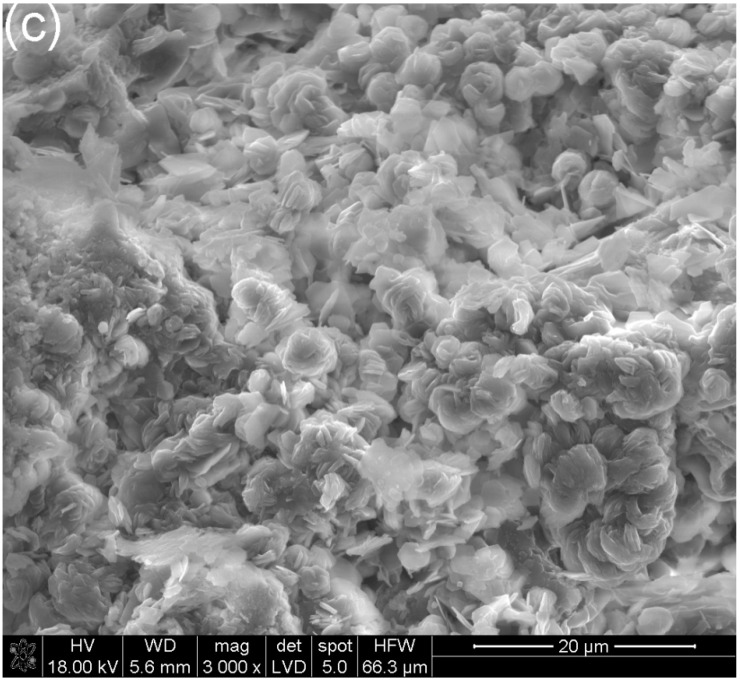
SEM image of the hydrated Sr_1.25_Ca_5.75_ZrAl_6_O_18_ cement (Sample A) at the age of 7 d (**a**). Spot 1 EDS analysis; (**b**) EDS spectrum of the sample in the microarea 1—Ca-rich (Sr,C)_3_AH_6_; (**c**) overview of microstructure.

Most of the C_3_AH_6_ or Ca-rich (Sr,C)_3_AH_6_ crystals formed after a hydration time of 7 d attain the shape of cubes, pyritohedra or other more complex forms of the isometric system, which are reinforced with Al(OH)_3_ crystals (Figure 6a,c). As observed before, those hydration products are strongly dependent on curing time and the Ca peak (Figure 6b) intensity increases relative to the Sr peak intensity (Figure 5b). Hence, as the curing time increases, the crystals belonging to a cubic or isometric system formed initially as a transient Sr-rich (Sr,C)_3_AH_6_ were replaced with Ca-rich (Sr,C)_3_AH_6_ or pure C_3_AH_6_. An unavoidable change of one form of calcium aluminate hydrate to another can be found elsewhere [32,33,34].

## 4. Conclusions

According to the current research, many conclusions can be drawn:

(1) The Sr-doped cement is developed through structural substitution for Ca ions by Sr ions in the Ca_7_ZrAl_6_O_18_ clinker phase.
(2) Strontium was used as a retarding agent to block this cement clinker phase hydration at a curing temperature of 50 °C. Hence, the residual unhydrated cement particles in the hardened Sr_1.25_Ca_5.75_ZrAl_6_O_18_ cement paste were present for much longer than for the undoped Ca_7_ZrAl_6_O_18_ clinker phase sample as it was observed by XRD.
(3) The hydration of both Ca_7_ZrAl_6_O_18_ and Sr_1.25_Ca_5.75_ZrAl_6_O_18_ cements was also inspected using the ^27^Al MAS NMR technique. This hydration is accompanied by a change of Al-coordination from tetrahedral to octahedral. This complete conversion from anhydrous ^27^Al^IV^ to hydrated ^27^Al^VI^ species was achieved during the first 24 h of hydration at 50 °C for Ca_7_ZrAl_6_O_18_ and during 7 d of hydration at 50 °C for Sr_1.25_Ca_5.75_ZrAl_6_O_18_.
(4) The hexagonal phases were formed starting in the very first minutes of hydration of these cements. For each cement type tested, these unstable hydrates consist mainly of C_4_AH_19_ and probably of C_2_AH_8_ as it was observed by XRD.
(5) The formation of a thermodynamically stable phase pure C_3_AH_6_ or Ca-rich (Sr,C)_3_AH_6_ in the hardened Sr_1.25_Ca_5.75_ZrAl_6_O_18_ cement paste was preceded by that of a number of less stable phases, i.e.,Sr-rich (Sr,C)_3_AH_6_ hydrate and other hexagonal Ca-Al hydrates. The Sr-rich (Sr,C)_3_AH_6_ hydrate existing between 0.5 h and 7 d of curing was isostructural with the Ca-rich (Sr,C)_3_AH_6_ or pure C_3_AH_6_ formed at the age of 7 d.
(6) The transformation of the hexagonal calcium aluminate hydrates and Sr-rich (Sr,C)_3_AH_6_ hydrate into the cubic phase Ca-rich (Sr,C)_3_AH_6_ or pure C_3_AH_6_ was expressed in terms of chemical shift from ca. 4 ppm to ca. 6 ppm in the hardened Sr_1.25_Ca_5.75_ZrAl_6_O_18_ cement paste at the age of 7 d. The same ^27^Al NMR chemical shift was detected at the age of 24 h for the reference hardened Ca_7_ZrAl_6_O_18_ cement paste.

## Figures and Tables

**Table 1:** Cement paste formulations, curing conditions and details of sample designation.

Sample Designation	Cement/Sample	Hydration Time	Water-to-Cement Ratio (w/c), Temperature
**A**	Sr_1.25_Ca_5.75_ZrAl_6_O_18_(as a solid solution)	15 min, 0.5 h, 1 h, 2 h, 3 h, 24 h, 48 h, 72 h, 7 d and 14 d	w/c = 1.0, T = 50 °C
**B**	SrAl_2_O_4_(as a reference undoped compound)	7 d	w/c = 0.5, T = 50 °C
**C**	Ca_7_ZrAl_6_O_18_(as a reference undoped compound)	15 min, 0.5 h, 1 h, 2 h, 3 h, 24 h, 48 h, 72 h, 7 d and 14 d	w/c = 1.0, T = 50 °C

## Nomenclature

C	CaO
A	Al_2_O_3_
Z	ZrO_2_
Sr	SrO
H	H_2_O
